# The role of extraversion and neuroticism for experiencing stress during the third wave of the COVID-19 pandemic

**DOI:** 10.1007/s12144-021-02600-y

**Published:** 2021-12-09

**Authors:** Jennifer A. Bellingtier, Marcus Mund, Cornelia Wrzus

**Affiliations:** 1 Department of Developmental Psychology, Friedrich Schiller University Jena, Am Steiger 3, Haus 1, 07743 Jena, Germany; 2 Department of Psychological Assessment and Personality Psychology, University of Klagenfurt, Klagenfurt, Austria; 3 Department of Psychological Aging Research, Heidelberg University, Heidelberg, Germany

**Keywords:** Stress process, Personality facets, Extraversion, Neuroticism, COVID-19, Transactional theory of stress

## Abstract

Although long postulated, it has been scarcely researched how personality traits play out differently in distinct situations. We examined if Neuroticism and Extraversion, personality traits known to moderate stress processes, function differently in highly stressful situations requiring reduced social contact, that is, the COVID-19 pandemic. Based on past findings, we expected neuroticism to be associated with exacerbated perceptions of stress. In contrast to past findings, we expected extraversion, which usually ameliorates stress, to be associated with intensified perceptions of stress, especially in regard to the sociability facet. During the third wave of the COVID-19 pandemic in Germany, one-hundred-thirty adults (age *M* = 21.7 years) reported on their personality traits including their facets with the BFI-2, COVID-19-related stressors, and their perceived stress during the last month (using the PSS). Findings indicated that neuroticism was associated with higher perceived stress regardless of the COVID-19-related stressors experienced. Facet level analysis revealed differences for anxiety, depression, and volatility. Importantly, trait extraversion was unassociated with stress experiences, whereas specifically the facet of sociability was associated with higher perceived stress. Also, the facets of assertiveness and energy both moderated the relationship between COVID-19-related stressors and perceived stress. In line with the transactional theory of stress, our findings indicate that perceptions of stress were best understood by looking at the interaction of environmental stressors and personality differences. Furthermore, the study substantiates that facets of personality traits offer unique information beyond broad traits in specific contexts.

During stressful events and severe crises, most people turn to others for support, connection, and belongingness (Scott et al., 2014). Accordingly, people higher in extraversion, who typically have larger social networks, experience less stress during stressful events (Carver & Connor-Smith, 2010). The COVID-19 pandemic might be a unique situation where, paradoxically, more extraverted people are more stressed due to reduced social contact during the pandemic, which is normally vital for them. Their stress responses may be more similar to those higher in neuroticism who tend to perceive greater stress during difficult times (Suls & Martin, 2005). Furthermore, as the pandemic wears on, the effects of continuous contact restrictions might pile up over time. In the middle of Germany’s third wave of the COVID-19 pandemic, we sought to investigate for whom the current situation is perceived as most stressful. We focus on individual differences in extraversion and neuroticism because both traits have been most consistently linked to affective experiences (Hughes et al., 2020).

## Transactional Theory of Stress

The transactional theory of stress (Lazarus & Folkman, 1987) conceives of stress as an interaction of situational and personal variables, which individuals appraise in regard to their personal relevance and their abilities to cope. The theory posits that the same stressful event will not be experienced in the same way, nor have the same outcomes, for all individuals. In other words, a stressor may be appraised, or perceived, differently depending on individual and situational circumstances. Differences in stress perceptions have implications for physical (e.g., cardiovascular disease, telomere length) and mental health (e.g., depression, suicidal behavior) outcomes (Cristóbal-Narváez et al., 2020; De Berardis et al., 2018; Schutte & Malouff, 2016; Vahedian-Azimi & Moayed, 2019). In order to fully capture the stress process, it is important to examine differences in the situational stressors experienced, individual differences in those experiencing the stressors, and variations in their appraisals of the experience. Here we examine how variations in the experience of COVID-19-related stressors and personality are associated with severity of stress appraisals.

## Personality and Stress

Prior research has established that personality differences modulate the stress process (Bolger & Zuckerman, 1995; Leger et al., 2016). The most consistent findings exist for neuroticism, characterized by depression, anxiety, and emotional volatility, which is associated with higher stress appraisals and greater reactivity to stressful events (Ebstrup et al., 2011; Leger et al., 2016). On the other hand, extraversion, characterized by sociability, assertiveness, and energy, has been typically associated with lower stress appraisals and less reactivity to stressful events, including negative affect (Leger et al., 2016; Strickhouser et al., 2017). However, the transactional theory of stress (Lazarus & Folkman, 1987) suggests that personal characteristics will not necessarily predict stress appraisals in the same fashion in all circumstances, but may function differently when the situational context varies. Here we consider if the situational context of the third wave of the COVID-19 pandemic in Germany may alter how some aspects of personality traits relate to stress appraisals.

### Neuroticism and COVID-19 Pandemic-Related Stress Experiences

Current evidence for the role of neuroticism in COVID-19 pandemic-related stress processes largely supports its function as an aggravator of the stress process. During the first wave of the COVID-19 pandemic in Germany, individuals higher in neuroticism were more likely to perceive the pandemic as strongly affecting their personal lives (Schmiedeberg & Thönnissen, 2021). Greater perceived stress and worry were also reported in the early stages of the COVID-19 pandemic for those high in neuroticism in France (Michinov & Michinov, 2021), Slovenia (Zager Kocjan et al., 2021), and Italy (Sebri et al., 2021). At the start of the COVID-19 pandemic, neuroticism predicted more emotion-focused coping in Israel (Agbaria & Mokh, 2021), higher levels of depression and anxiety symptoms in Germany, India, Israel, and the United States (Lee et al., 2020; Nudelman et al., 2021), lower well-being in Switzerland (Gubler et al., 2021), and higher levels of COVID-related anxiety in the United States (Nikčević et al., 2021). Given the consistency of the neuroticism effects through the first wave of the COVID-19 pandemic, we anticipate that high levels of neuroticism will continue to predict higher stress appraisals in the third wave of the pandemic in Germany.

### Extraversion and COVID-19 Pandemic-Related Stress Experiences

Extraversion has typically played a protective role in the stress process, and some research from early in the COVID-19 pandemic supports this function. For example, individuals from the United States reported less anxiety, depression, and COVID-related concerns when they were higher in extraversion (Nikčević et al., 2021). Likewise, higher levels of extraversion protected against COVID-related worry in Italian adults (Sebri et al., 2021) and were associated with beneficial problem-focused coping in an Israeli sample (Agbaria & Mokh, 2021). However, other findings have been more equivocal. For example, German participants reported being more affected by the first wave of the COVID-19 pandemic if they did not have a partner *and* were high in extraversion (Schmiedeberg & Thönnissen, 2021). Findings from Switzerland, Germany, and Slovenia suggested that in the context of COVID-19, higher extraversion was actually associated with higher loneliness and poorer well-being (Alt et al., 2021; Entringer & Gosling, 2021; Gubler et al., 2021; Zager Kocjan et al., 2021). The social contact restrictions of the COVID-19 pandemic may be particularly hard on more extraverted individuals, with a large study across 55 countries indicating that higher extraversion was associated with being less likely to stay at home in the first wave of the pandemic (Götz et al., 2021). We thus examined, whether extraverted individuals may be particularly challenged during later phases of the COVID-19 pandemic when social contact has been restricted for months.

Given pre-pandemic research supporting the beneficial role of extraversion in the stress process, the unique contact restrictions of the COVID-19 pandemic, and the mixed post-pandemic findings, we sought a more nuanced perspective on extraversion by examining facet specific effects (Smillie et al., 2013, 2015; Soto & John, 2017). The Big Five traits are very broad and pertain to very general dispositions, whereas their facets (e.g., sociability, assertiveness, and energy as facets of Extraversion) are narrower in scope and pertain to more specific aspects of affect, cognition, and behavior (McCrae & Costa, 2008; Soto & John, 2017). Due to their higher specificity, it is possible that the facets of a Big Five trait show diverging associations with an outcome. Indeed, such differential associations have been found frequently (e.g., Ashton, 1998; Mund & Neyer, 2014) and, thus, help to draw a nuanced and differentiated picture of how personality characteristics are associated with individual and interpersonal consequences. In the present study, we consider three facets of extraversion: sociability (i.e., the tendency to be outgoing and sociable), assertiveness (i.e., the tendency to be dominant and take charge), and energy (i.e., the tendency to be active and enthusiastic; Soto & John, 2017). We predicted that sociability would be associated with greater perceived stress, as compared to assertiveness and energy. We reasoned that the COVID-19 pandemic-related social restrictions would be most detrimental to those high in the sociability facet leading to higher stress appraisals, whereas assertiveness and energy would be less influenced by the pandemic situation.

## Current Study

In the current study, we sought to understand how the experience of COVID-19-related stressors interacted with personality to predict stress appraisals during the third wave of the COVID-19 pandemic in Germany in a sample of young adults. We preregistered the following hypotheses:

- (H1) Individuals high in neuroticism will perceive greater stress and experience more negative outcomes when COVID-19 stressors are high than those low in neuroticism.
- (H2) Individuals high in extraversion will perceive greater stress and experience more negative outcomes when COVID-19 stressors are high than those low in extraversion.
- (H3) Individuals high in sociability will perceive greater stress and experience more negative outcomes when COVID-19 stressors are high than those high in assertiveness and/or in energy.^1^

## Method

### Participants and Procedure

Participants were 130 adults ranging in age from 19 to 40 years (*M* = 21.73, *SD* = 3.57; 22% men) who agreed to participate in an online survey in exchange for either a gift card raffle or partial course credit. Inclusion criteria included being at least 18 years of age as well as speaking German. Exclusion criteria included incorrectly answering at least two of three attention check questions and taking less than two minutes to complete the survey. Twenty-three additional participants completed at least part of the survey but were not included in these analyses: four were excluded for failing at least two of these three attention checks, nine were excluded for spending less than two minutes on the survey, and ten were excluded for not providing responses to the variables of interest in this study. Sample size was determined by an a priori power analysis for linear multiple regression specifying a power of .80 to detect a small (f^2^ = .15) effect at alpha < .05 with 11 predictors using G*Power (Faul et al., 2007).

After completing an informed consent, participants completed survey measures to assess personality traits, their experience of COVID-19-related stressors, and perceived stress during the last month. Surveys were completed during a 2-week timespan from the 5th to 18th of May, 2021. During this time, Germany was experiencing the third wave of the COVID-19 pandemic. Most states were in a federally imposed lockdown as stipulated by the Infection Protection Act, which took effect on the 24th of April, 2021. This act limited the authority of the individual German states when 7-day incidence values exceeded certain thresholds and imposed federally mandated contact restrictions, curfews, closing of non-essential shops, and the closure of schools. The majority of university classes were held online and employees were requested to work from home when possible. At this point, access to vaccines was limited to older adults and high risk groups.

The study was approved by the ethics committee at Friedrich Schiller University Jena (FSV 21/013).

### Measures

#### Personality

Personality was assessed using the German version of the Big Five Inventory-2 (BFI-2, Danner et al., 2019; Soto & John, 2017). The inventory assesses the Big Five traits (12-items per trait), which can be distinguished into three facets per trait (4-items per facet, Soto & John, 2017). Participants were asked to indicate if each item described themselves on a 5-point scale ranging from *disagree strongly* to *agree strongly* (coded as 1 to 5). Participants received a mean score for each trait and facet.

#### COVID-19-Related Stressors

COVID-19-related stressors were assessed with the first 8-items of the Coronavirus Impact Scale (Stoddard & Kaufman, 2020) translated into German, where participants rate how the pandemic has changed different life domains (e.g., income, access to medical care, social relationships) on a 0 (*no change*) to 3 (*severe change*) scale. We added four similarly-worded items to assess change related to concern for others, interactions with colleagues, workload, and important life events. A mean score was created for each participant.

#### Stressor Appraisals

Participants were asked to indicate how stressful they perceived the last month of the pandemic to be using the 10-item German translation of the Perceived Stress Scale (Cohen et al., 1983; Reis et al., 2019). The scale includes items tapping primary appraisals (e.g., “In the last month of the pandemic, how often have you felt nervous and ‘stressed’?”) as well as secondary appraisals (e.g., “In the last month of the pandemic, how often have you found that you could not cope with all the things that you had to do?”). Items are answered on a 5-point scale ranging from *never* to *very often* (coded as 1 to 5), and averaged to create the scale mean.

### Analytic Strategy

We considered any scores ± 2.5 standard deviations from the mean as an outlier and winsorized the score to the next valid score.^2^

To examine the relationship between COVID-19-related stressors, personality, and their interaction with stress appraisals, we computed a series of bias-corrected and accelerated bootstrapped hierarchical regressions with SPSS version 25. All predictor variables were grand-mean centered. Variables were entered in three steps: (1) COVID-19-related stressors, (2) personality trait(s) or facet(s), and (3) the interaction between the variables specified in steps one and two. In this way we were able to examine the additional variance in stressors appraisals explained by personality. We analyzed each trait separately. The three facets of each trait were modeled together. Results of the hypotheses-based analyses for Extraversion and Neuroticism are reported in the main text; results of exploratory analyses for the other three traits are reported in the supplementary materials.

## Results

Descriptive statistics are presented in Table 1. In each model we began by entering COVID-19-related stressors as the sole predictor of stressor appraisals. Individuals who experienced more COVID-19-related stressors reported higher levels of perceived stress in the last month (β = .49, *p* < .001, *R*^2^ = .24).

**Table 1:** Descriptive Statistics for Study Variables

Variables	*M* (*SD*)	Range	ω	1	2	3	4	5	6	7	8	9
1. Perceived Stress	2.99 (0.69)	1.40–4.40	.88									
2. C19 Stressors	2.11 (0.37)	1.42–3.00	.77	.49*								
3. Neuroticism	2.86 (0.73)	1.17–4.50	.91	.71*	.35*							
4. Anxiety	3.21 (0.77)	1.50–4.75	.75	.62*	.27*	.90*						
5. Depression	2.68 (0.86)	1.00–4.75	.85	.67*	.35*	.88*	.69*					
6. Volatility	2.70 (0.83)	1.00–4.75	.82	.59*	.31*	.89*	.73*	.64*				
7. Extraversion	3.34 (0.63)	1.50–5.00	.88	−.09	.06	−.23*	−.23*	−.33*	−.04			
8. Sociability	3.34 (0.85)	1.50–5.00	.84	.08	.08	−.08	−.10	−.18*	.08	.89*		
9. Assertiveness	3.15 (0.74)	1.00–5.00	.78	−.27*	−.02	−.31*	−.31*	−.39*	−.13	.83*	.60*	
10. Energy	3.55 (0.65)	2.00–5.00	.70	−.08	.08	−.20*	−.20*	−.29*	−.07	.81*	.59*	.50*

*Note.* ω = omega, internal consistency reliability

*p < .05

### Neuroticism

**Fig. 1:**
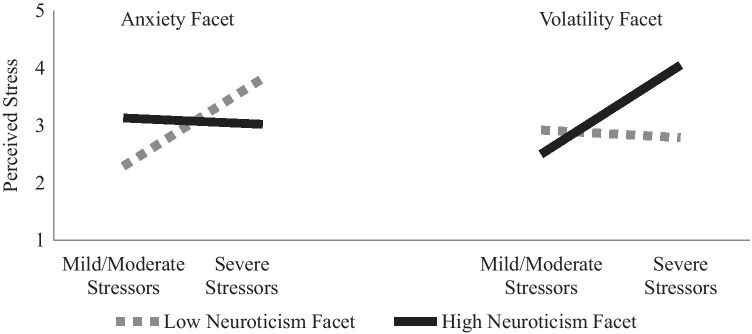
Interaction of Neuroticism Facets and COVID-19-Related Stressors Predicting Perceived Stress. *Note.* Mild/Moderate stressors are graphed at 1.5, and severe stressors at 3.0, on the coronavirus impact scale. The neuroticism facets, anxiety and volatility, are graphed at ± 1 *SD*. Simple slopes for low anxiety = 1.02, *p* = .008, for high anxiety = −0.07, *p* = .867, low volatility = −0.09, *p* = .836, high volatility = 1.03, *p* = .038

At the trait level, higher neuroticism was associated with greater perceived stress regardless of the COVID-19-related stressors experienced (see Table 2). At the trait level it explained 33% of the variance in perceived stress and 38% at the facet level. Higher anxiety was associated with higher perceived stress in Step 2, and this was qualified by a significant anxiety-by-stressor interaction in Step 3. Individuals low in anxiety reported more perceived stress when COVID-19-related stressors were higher, versus lower, whereas those high in anxiety reported similar levels of perceived stress regardless of the COVID-19-related stressors experienced (see left side Fig. 1). The depression facet was associated with higher perceived stress regardless of the COVID-19-related stressors experienced. Higher volatility was associated with greater perceived stress when COVID-19-related stressors were high, versus low, whereas those low in volatility did not vary their stress appraisals when COVID-19-related stressors were low or high (see right side Fig. 1).

**Table 2:** Predicting Perceived Stress from Neuroticism and Its Facets

Variables	Trait		Facets	
	B [95% BCa CI]	β	B [95% BCa CI]	β
**Step 1:**				
Intercept	2.98 [2.88, 3.09]		2.99 [2.89, 3.10]	
C19 Stressors	0.91 [0.64, 1.20]	.49*	0.91 [0.63, 1.15]	.49*
R^2^	.24*		.24*	
**Step 2:**				
Intercept	2.99 [2.89, 3.06]		2.99 [2.90, 3.08]	
C19 Stressors	0.51 [0.29, 0.73]	.28*	0.50 [0.28, 0.72]	.27*
Neuroticism	0.58 [0.47, 0.68]	.61*		
Anxiety			0.20 [0.05, 0.37]	.22*
Depression			0.28 [0.12, 0.42]	.35*
Volatility			0.11 [−0.08, 0.29]	.13
R^2^	.57*		.58*	
R^2^Δ	.33*		.34*	
**Step 3:**				
Intercept	3.00 [2.91, 3.09]		3.00 [2.91, 3.11]	
C19 Stressors	0.52 [0.32, 0.75]	.28*	0.47 [0.22, 0.71]	.26*
Neuroticism	0.58 [0.47, 0.68]	.61*		
Anxiety			0.11 [−0.05, 0.29]	.13
Depression			0.30 [0.15, 0.45]	.38*
Volatility			0.16 [−0.01, 0.32]	.19*
C19 Stressors x Neuroticism	−0.08 [−0.42, 0.21]	−.03		
C19 Stressors x Anxiety			−0.70 [−1.23, −0.14]	−.25*
C19 Stressors x Depression			−0.21 [−0.62, 0.17]	−.09
C19 Stressors x Volatility			0.67 [0.26, 0.98]	.30*
R^2^	.57*		.62*	
R^2^Δ	.00		.04*	

*Note.* Regressions run with 1000 Bias-Corrected Bootstrap Samples. C19 Stressors and neuroticism and its facets are grand mean centered

*p < .05

### Extraversion

**Fig. 2:**
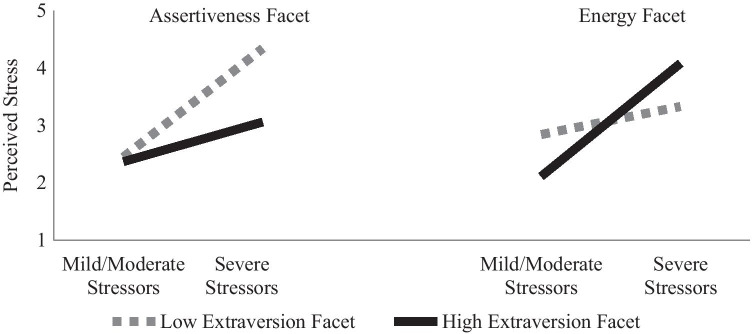
Interaction of Extraversion Facets and COVID-19-Related Stressors Predicting Perceived Stress. *Note.* Mild/Moderate stressors are graphed at 1.5, and severe stressors at 3.0, on the coronavirus impact scale. The extraversion facets, assertiveness and energy, are graphed at ± 1 *SD*. Simple slopes for low assertiveness = 1.20, *p* = .004, for high assertiveness = 0.44, *p* = .307, low energy = 0.32, *p* = .407, high energy = 1.31, *p* = .002

At the trait level, neither extraversion nor its interaction with COVID-19-related stressors explained additional variance in stressor appraisals (see Table 3). However, at the facet-level, extraversion explained an additional 18% of the variance in stressor appraisals. Higher sociability was associated with higher perceived stress in the last month regardless of the COVID-19-related stressors experienced. In contrast, higher assertiveness was associated with lower perceived stress regardless of the COVID-19-related stressors experienced, whereas lower assertiveness was associated with higher perceived stress, which was exacerbated for those who experienced more COVID-19-related stressors (see left side Fig. 2). The energy facet moderated the relationship between COVID-19-related stressors and perceived stress, such that those high in energy perceived more stress when COVID-19-related stressors were high, versus low, but those low in energy had similar stress perceptions regardless of the COVID-19-related stressors experienced (see right side Fig. 2).

**Table 3:** Predicting Perceived Stress from Extraversion and Its Facets

Variables	Trait		Facets	
	B [95% BCa CI]	β	B [95% BCa CI]	β
**Step 1:**				
Intercept	2.98 [2.88, 3.09]		2.99 [2.88, 3.09]	
C19 Stressors	0.91 [0.64, 1.20]	.49*	0.91 [0.63, 1.19]	.49*
R^2^	.24*		.24*	
**Step 2:**				
Intercept	2.99 [2.89, 3.09]		2.99 [2.88, 3.10]	
C19 Stressors	0.92 [0.66, 1.20]	.50*	0.86 [0.59, 1.10]	.46*
Extraversion	−0.13 [−0.29, 0.03]	−.12		
Sociability			0.29 [0.13, 0.45]	.36*
Assertiveness			−0.38 [−0.54, −0.23]	−.41*
Energy			−0.13 [−0.36, 0.08]	−.12
R^2^	.25*		.37*	
R^2^Δ	.02		.13*	
**Step 3:**				
Intercept	2.99 [2.88, 3.10]		2.98 [2.88, 3.07]	
C19 Stressors	0.92 [0.67, 1.20]	.50*	0.82 [0.57, 1.04]	.44*
Extraversion	−0.14 [−0.29, 0.03]	−.12		
Sociability			0.27 [0.09, 0.47]	.33*
Assertiveness			−0.41 [−0.56, −0.26]	−.43*
Energy			−0.10 [−0.31, 0.10]	−.09
C19 Stressors x Extraversion	−0.02 [−0.54, 0.41]	−.01		
C19 Stressors x Sociability			−0.18 [−0.65, 0.32]	−.08
C19 Stressors x Assertiveness			−0.52 [−0.92, 0.03]	−.19*
C19 Stressors x Energy			0.77 [0.34, 1.13]	.27*
R^2^	.25*		.42*	
R^2^Δ	.00		.05*	

*Note.* Regressions run with 1000 Bias-Corrected Bootstrap Samples. C19 Stressors and extraversion and its facets are grand mean centered

*p < .05

## Discussion

In line with the transactional theory of stress (Lazarus & Folkman, 1987), our findings indicate that perceptions of stress varied based on individuals’ personalities and the interaction of their personalities with COVID-19-related stressors. At the trait level, individuals higher in neuroticism reported higher perceived stress. Although extraversion was unrelated to stress at the trait level, at the facet level unique patterns emerged. Those high in sociability were more likely to report higher perceived stress regardless of the COVID-19-related stressors they experienced whereas high assertiveness and low activity interacted with COVID-19-related stressors to buffer stress perceptions. Importantly, our findings suggest that when seeking to understand the stress process within particular contexts it may be especially important to consider not just broad personality traits, but the specific facets of which they are composed (Ashton, 1998; Mund & Neyer, 2021).

### Neuroticism and Higher Stress Perceptions

In line with past research (Ebstrup et al., 2011; Leger et al., 2016), neuroticism was associated with greater subjective perceptions of stress during the last month of the COVID-19 pandemic. Both individuals high in trait-level neuroticism and facet-level depression reported more perceived stress regardless of the COVID-19-related stressors reported. The depression facet is characterized by higher negative affect, which could lead individuals to appraise situations more negatively irrespective of differences in the COVID-19-related stressors experienced. These findings align with other COVID-19-related research finding higher perceived stress and worry for those higher in neuroticism (Michinov & Michinov, 2021; Sebri et al., 2021; Zager Kocjan et al., 2021).

Although we had not hypothesized facet-level differences, our findings indicated that those lower in anxiety and higher in volatility perceived more stress when COVID-19-related stressors were higher, as compared to lower, whereas people with high anxiety and low volatility reported more stable (high) stress perceptions. In considering these findings, it should be noted that the facets were analyzed simultaneously, thus people’s standing on the other facets is controlled in these analyses.

For anxiety, individuals high in this facet do not appear to show additional reactivity to COVID-19-related stressors. One possibility is that these individuals expect the world to be an anxious, tense place, and thus the COVID-19 pandemic conditions align with their pre-existing expectations (Spielberger & Reheiser, 2009). Prior experiences with these types of situations could have a steeling effect for these individuals, which helps them to manage their stress (Höltge et al., 2018). On the other hand, those lower in anxiety but experiencing many COVID-19-related stressors, may be experiencing a more unusual or unexpected situation for themselves and are thus more reactive to the high onslaught of stressors.

Regarding volatility, this facet directly assesses individual’s tendency to be reactive to negative and stressful circumstances, thus it follows that these individuals would be more reactive to high levels of COVID-19 related stressors. Limited past research indicates that the facets of neuroticism may function differently in some stressful situations (e.g., workplace harassment; McCord, 2021). Future research that examines the facets in other stressful contexts is necessary to determine if this pattern is applicable beyond the specific context investigated here.

### Extraversion Varies by Facet

Although extraversion has previously been viewed as an asset for ameliorating the stress process (Leger et al., 2016; Strickhouser et al., 2017), we found that in the context of the COVID-19 pandemic, a more nuanced picture emerged. When considered at the trait level, there was no relationship between extraversion and perceived stress. However, analysis at the facet level indicated that this was likely due to the facets working in opposition to each other. Specifically, we found that individuals high in sociability perceived the last month as more stressful than those lower in sociability. In contrast, assertiveness showed the opposite pattern: those high in assertiveness perceived less stress than those lower in this facet of extraversion.

We had hypothesized that the sociability facet would be more likely to exacerbate the stress process than assertiveness or energy. Individuals high in this facet tend to be outgoing and enjoy interacting with others, which has become more difficult in the context of COVID-19 pandemic contact restrictions and physical distancing guidelines (Götz et al., 2021). Our findings indicated that these individuals perceived the last month to be more stressful regardless of the COVID-19-related stressors they experienced. This may be due to the breadth of this measure, which assessed COVID-19-related stressors beyond those related to contact restrictions (e.g., changes in income and healthcare access). These findings complement those of Zager Kocjan and colleagues (2020) who found extraversion to be associated with higher levels of perceived stress in the context of the COVID-19 pandemic and qualify them by pointing to sociability as the facet of extraversion most likely to amplify perception of stress.

On the other hand, the assertiveness facet of extraversion was associated with lower stress perceptions, and buffered stress perceptions when COVID-19-related stressors were high. Individuals high in assertiveness behave more dominantly and prefer to take charge of situations. These attributes could assist them in adopting active coping techniques that allow them to better navigate the COVID-19 pandemic (Agbaria & Mokh, 2021). Indeed, past research suggests that those who feel more in control of situations are less reactive to stressful events (Neupert et al., 2007). This finding complements the COVID-19-related findings suggesting extraversion can be an asset during the pandemic (Nikčević et al., 2021; Sebri et al., 2021) and qualifies them by indicating that the beneficial effects of extraversion may be most attributable to the assertiveness facet.

The third extraversion facet, energy, did not have a main effect on perceptions of stress, but moderated perceptions depending on the level of COVID-19-related stressors experienced (cf. Schmiedeberg & Thönnissen, 2021). Compared to those low in energy, those high in energy reported lower perceived stress when COVID-19-related stressors were low, but higher perceived stress when COVID-19-related stressors were high. People high in energy are enthusiastic, excited, and active, and might have eagerly embraced a new hobby or found a new way to engage with others online during the pandemic. On the other hand, if these individuals encountered numerous stressors related to the pandemic their energy may have needed to be directed at these stressors (e.g., becoming a homeschool teacher, finding a new job, etc.). Here we see that more objective differences in the COVID-19-related stressors these individuals encountered likely altered their stress perceptions depending upon how their energy was used.

### Limitations and Future Directions

The COVID-19 pandemic has been a uniquely stressful situation across the globe (Kowal et al., 2020). Our study captures a unique moment in this pandemic--the third wave, in a unique population--a modest sample of younger German adults, in a unique context—the reoccurring federally imposed lockdown prior to the wide-availability of vaccines. Thus the generalizability of our findings is limited by these considerations. More specifically, individuals from other age-groups or countries, as well as those experiencing stress at other points in the pandemic, may show different associations between personality and stress perceptions. We believe a full understanding of these processes will only be possible by synthesizing numerous studies from many different populations and time-points in the COVID-19 pandemic. We have embedded our findings within the existing research, but we expect that as contact restrictions wax and wane, vaccine availability expands, and novel variants emerge, new findings will appear that will allow us to further contextualize the current results.

Future research could consider how these findings manifest for more distal outcomes of the stress process, for example, life-satisfaction and mental health (Orsolini et al., 2020; Prati & Mancini, 2021). Furthermore, as our study addresses a single moment in time and relies on self-report measures, the incorporation of longitudinal and physiological measures would help to further expand on our understanding of the stress process during the COVID-19 pandemic.

## Conclusions

In conclusion, our findings complement and extend the current research on the role of personality for the stress process during the COVID-19 pandemic. Most importantly, by adopting a facet level approach, we were able to offer insight into the functioning of neuroticism and clarify the mixed findings regarding extraversion during the COVID-19 pandemic. In particular, we demonstrated that some facets of personality function in counter-typical fashion during the pandemic: sociability no longer buffered, but enhanced stress perceptions whereas anxiety no longer augmented, but ameliorated them. One implication of this pattern is that those who are typically most at risk for negative clinical outcomes may not be those who suffer most from the pandemic. Furthermore, the findings indicate the potential for negative mental, not just physical, health outcomes as a result of the COVID-19 pandemic and measures to reduce its impact.

Our findings support the importance of simultaneously considering environmental and personal differences for understanding the stress process (Caspi & Moffitt, 1993; Lazarus & Folkman, 1987; Neupert et al., 2021). We encourage future researchers interested in understanding specific stressful situations to go beyond broad traits and consider the role of finer-grained personality facets.

## Data Availability

The dataset generated during and analyzed during the current study are available in the OSF repository for this study, https://osf.io/eqwtg/?view_only=2dd67a557e5d496a8747b6bfc481f100

## Footnotes

1 The project also focused on other research questions/hypotheses, which are not covered in the current manuscript. In the preregistration *energy* is referred to as *activity,* and *COVID-19* stressors are referred to as *corona* stressors.

2 At most, 3% of scores for a given variable were winsorized. The following number of cases were effected: 1 COVID-19 stressor, 1 depression, 1 volatility, 1 activity, 1 agreeableness, 4 compassion, 1 respectfulness, 3 conscientiousness, 2 organization, 4 responsibility, 1 openness, 2 aesthetic sensitivity, 2 curiosity, and 1 creativity.
